# Differential relationship of observer-rated and self-rated depression and anxiety scales with heart rate variability features

**DOI:** 10.3389/fpsyt.2023.1124550

**Published:** 2023-04-03

**Authors:** Jinsil Ham, Hesun Erin Kim, Jae-Jin Kim, Jeong-Ho Seok, Eunjoo Kim, Jin Young Park, Boreom Lee, Jooyoung Oh

**Affiliations:** ^1^Department of Biomedical Science and Engineering (BMSE), Gwangju Institute of Science and Technology (GIST), Gwangju, Republic of Korea; ^2^Institute of Behavioral Sciences in Medicine, Yonsei University College of Medicine, Seoul, Republic of Korea; ^3^Department of Psychiatry, Gangnam Severance Hospital, Yonsei University College of Medicine, Seoul, Republic of Korea; ^4^Department of Psychiatry, Yongin Severance Hospital, Yonsei University College of Medicine, Yongin, Republic of Korea

**Keywords:** heart rate variability, clinician-rated assessment, self-rated questionnaires, depression, anxiety, vagal tone

## Abstract

Heart rate variability (HRV) is a known psychophysiological marker for diverse psychiatric symptoms. In this study, we aimed to explore the potential for clinical use of HRV by investigating the interrelationship between HRV indices and clinical measures mainly used to assess depressive and anxious symptoms. Participants who reported depressive and anxious symptoms were designated into the following groups: group 1, clinician-rated and self-rated depression; group 2, only self-rated depression; group 3, clinician-rated and self-rated anxiety; group 4, only self-rated anxiety. Statistical comparisons were performed between these groups to investigate the association between HRV and clinical measures. As a result, HRV variables showed significant correlations only with the clinician-rated assessments. Moreover, both time and frequency domain HRV indices were significantly different between groups 1 and 2, but groups 3 and 4 showed significant differences only in frequency domain HRV indices. Our study showed that HRV is an objective indicator for depressive or anxious symptoms. Additionally, it is considered a potential indicator for predicting the severity or state of depressive symptoms rather than of anxious symptoms. This study will contribute to increasing the diagnostic utility of discriminating those symptoms based on HRV in the future.

## 1. Introduction

In the 21st century, mental disorders are considered one of the most serious diseases that threaten people’s health and life (1). Many people worldwide are suffering from various mental disorders, with a 12 month prevalence of 17.6% and lifetime prevalence of 29.2% (2). This prevalence has not only significantly increased during the last decade (2–4) but also continues to increase due to circumstances such as the COVID-19 pandemic (5, 6). Mental disorders are one of the leading causes of the global health-related burden (5), imposing a substantial financial strain on the medical system with growing negative social and economic consequences (6, 7). Among the common symptoms of mental disorders, depression and anxiety are associated with emotional distress of patients, symptom severity, prognosis, and recurrence of related disorders (8), while also being the most prevalent complications of psychiatric disorders (9). Therefore, accurate and reliable detection of depressive or anxious symptoms can enable the early detection, intervention, and treatment of psychiatric disorders.

For the most part, psychiatric symptoms are assessed through clinician-rated or self-rated questionnaires in many clinics. The clinician-rated questionnaire is valid and reliable to objectively assess the severity of symptoms; however, it requires complex, time-consuming, and costly endeavors (10, 11). Conversely, although the self-rated questionnaire is an efficient way to save time and cost, the total scores can be over- or under-estimated depending on the character, predisposition, and cognitive ability of the respondents (10, 12), which is a serious problem. There is a controversy on the use of these clinical measures. Several previous studies reported that the severity of depression or anxious symptoms evaluated by both types of measurement was highly concordant, suggesting the interchangeability with each other (13–16). However, some previous studies suggested that evaluation results by those measures showed significant discrepancies, even asserting that only clinician-rated measures have statistical advantages to evaluate the severity of depressive or anxious symptoms (10, 17–19). Contrarily, it was also suggested that both clinician-rated and self-rated measures should be utilized together rather than using a single measure because each measurement provides unique information relevant to clinical prognosis (20). These discrepancies in opinion may result from the following reasons: clinicians’ or patients’ biases in interpreting symptomatology, limited insight of clinicians or patients, cognitive functioning and/or personality traits of patients, and patient demographic characteristics (such as age, sex, education, and occupation) (12, 15, 18). In essence, there are still no definitive and gold-standard assessments to quantify the severity of depressive or anxious symptoms. There is a need for an alternative method that can measure the severity of depression or anxiety in a more stable and reliable way by compensating for the above mentioned limitations of scale-based assessments.

Conversely, various heart rate variability (HRV) indices are considered more objective metrics for describing the health of the body and mind (21, 22). HRV, defined as complex beat-to-beat variations between heartbeats, is regulated by the interaction of the sympathetic and parasympathetic (vagal) components of the autonomic nervous system (ANS) and is recognized as a psychophysiological marker that reliably, objectively, and indirectly reflects the functioning of the ANS (23–25). In particular, dysregulation of the ANS can be quantified using HRV in diverse psychiatric symptoms and disorders (24, 26), and there have been numerous attempts to explore the relationship between the symptoms of depression and anxiety and HRV (27–29). Reduction in HRV is known to be a general indicator for patients with psychiatric disorders rather than healthy controls (30), and this finding is also shown in patients with depressive or anxious symptoms. Accordingly, meta-analyses, which provide more convincing evidence, also showed that reduced HRV is associated with both depression and anxiety. Anxious symptoms might relate to failure to inhibition of cognitive, behavioral, and affective responses, which reduce vagal function and eventually lead to decreased HRV indices (e.g., high-frequency power (HF), standard deviation of all RR intervals (SDRR), and root mean square of successive RR interval differences (RMSSD)) (27). Likewise, depressive symptoms might relate to somatomotor deficits which lead to changed HRV indices such as decreased HF and RMSSD, and increased low-frequency power (LF) and low- to high-frequency ratio (LF/HF) (29). Particularly, Kemp et al. showed that depressive symptoms had a significant negative correlation with HRV in major depressive disorder (MDD) patients, suggesting that HRV can be reflected in the severity of depressive symptoms (29). These findings support that HRV is not only an objective and quantitative psychophysiological marker but also has the potential for screening psychiatric symptoms and disorders.

It is necessary to directly compare the autonomic balance indicator—HRV—with the clinical assessments frequently used for evaluating various psychiatric symptoms, in order to support or increase the possibility of symptom evaluations based on HRV in clinics. A few studies have found that HRV indices have significant correlations with clinician-rated (31–34) and self-rated measures (35) and therefore have the capability to discriminate depressive symptoms in MDD patients. In the case of anxiety, a study demonstrated found no significant correlation between self-rated measures and HRV indices in patients with anxious symptoms (36). However, Bilgin et al. also examined the relationship between the frequency domain measures of HRV and both clinician-rated and self-rated assessments used to determine anxiety levels and identified a significant relationship between the frequency domain HRV indices and the outcomes of those clinical measures (37). Although several studies have been conducted to date, few studies have simultaneously compared the relationship between HRV indices and both clinician-rated and self-rated questionnaires for depressive or anxious symptoms. In other words, existing studies have limitations related to exploring the relationship between HRV indices and using only clinician-rated or self-rated questionnaires, applying only specific domain measures of HRV, or designating the participants who have specific mental disorders. To compensate for these limitations, a more systematic study is needed to precisely examine the relationship between HRV indices and both clinician-rated and self-rated assessments, with respect to the transdiagnostic dimensional approach (38).

As mentioned above, the use of an appropriate measure is required to investigate depressive and anxious symptoms accurately. However, which of the two clinical measures, clinician-rated and self-rated questionnaires, could better reflect the severity of depressive and anxious symptoms is somewhat questionable since each has clear advantages and disadvantages. Moreover, although HRV is considered an objective psychophysiological marker, it is still difficult to be used independently for discriminating the symptoms of depression and anxiety due to the lack of evidence about its relationship with psychological assessments. Therefore, it is required to investigate the interrelationship between physiological changes (i.e., HRV), clinician-rated assessments, and self-rated questionnaires by acquiring and comparing them at the same time. Eventually, it is expected to shed light on the clinical application of HRV as well as examine whether each HRV index is relevant to the objective or subjective clinical measures. Furthermore, a more reliable, objective, and quantitative method can be obtained for identifying depressive and anxious symptoms.

In this study, we hypothesized that the HRV indices would have a significant interrelationship with clinical measures. In particular, as the HRV indices were obtained based on bio-signals, they would show higher correlations with the results of the clinician-rated measure than those of the self-rated measure, which may signify that the HRV indices have objective rather than subjective implications. For this purpose, we simultaneously evaluated depressive or anxious symptoms of patients using clinical-rated and self-rated questionnaires, and acquired electrocardiogram (ECG) data from the patients to extract various HRV indices. Thereafter, the interrelationship among the HRV indices, the clinician-rated assessments, and the self-rated questionnaire was determined.

## 2. Materials and methods

### 2.1. Participants

**Figure 1:**
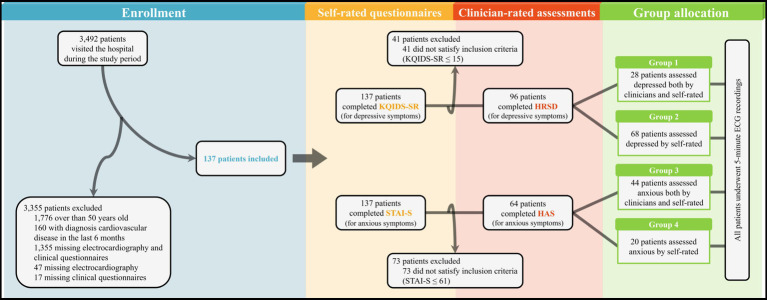
Flow diagram of participants’ enrollment, assessment, and allocation. KQIDS-SR, Korean Quick Inventory of Depressive Symptomatology Self-Report; STAI-S, State–Trait Anxiety Inventory-State; HRSD, Hamilton Rating Scale for Depression; HAS, Hamilton Anxiety Scale; ECG, Electrocardiogram.

Participants were outpatients from the psychiatric department of Yonsei University Gangnam Severance Hospital (Seoul, South Korea) who reported depressive and/or anxious symptoms from November 2018, to January 2022. During this period, a total of 3,492 new patients visited the psychiatric outpatient clinic of the hospital, regardless of the diagnosis. As shown in Figure 1, most patients were excluded at the first stage according to the following exclusion criteria: (i) patients aged over 50 with a high chance of prevalence of cardiovascular disease (39), (ii) patients diagnosed with a cardiovascular disease within the past 6 months, and (iii) patients who did not complete ECG assessments and/or clinical questionnaires. Additionally, patients who were unable to complete the self-rated questionnaires—such as patients with intellectual disability, cognitive disorders such as dementia, or severe active symptoms of psychotic disorders that cause problems in reality testing— were also excluded. After the first exclusion, 137 patients were evaluated for depressive and anxious symptoms using self-rated questionnaires and clinician-rated assessments. The Korean Quick Inventory of Depressive Symptomatology Self-Report (KQIDS-SR) and State–Trait Anxiety Inventory-State (STAI-S) were used to measure the severity of depressive and anxious symptoms, respectively. To include only patients with obvious symptoms of depression and/or anxiety, 41 patients with total KQIDS-SR scores (40) of ≤15 and 73 patients with total STAI-S scores (41) of ≤61 were excluded. After applying the exclusion criteria, clinician-rated assessments were used for further subdivision. The Hamilton Rating Scale for Depression (HRSD) (42, 43) and Hamilton Anxiety Scale (HAS) (44, 45) were used for patients with symptoms of depression (*n* = 96) and anxiety (*n* = 64), respectively. When the total of scores of HRSD or HAS were 24 or higher, patients were considered to have overt symptoms of depression or anxiety. Among the patients excluded based on the self-rated questionnaire results, 40 and 55 patients were not depressed or anxious, respectively, and 1 and 18 patients were considered to have depressive or anxious symptoms only by clinicians, respectively. More detailed descriptions of the group allocation according to the results of clinical measures are provided in Supplementary Tables S1 and S2. All clinical data were retrospectively collected from the Severance Hospital’s Electronic Medical Record system, and informed consent was not obtained. This retrospective study protocol was approved by the institutional review board of Gangnam Severance Hospital (3–2022-0009).

### 2.2. Assessment of depression and anxiety

Several clinical assessments were conducted to investigate the severity of depression and/or anxiety in participants. Self-rated questionnaires, such as KQIDS-SR and STAI-S, were used to measure the degree of subjective depression and/or anxiety states. The KQIDS-SR (40) has 16-items that are designed to measure symptoms of depression, including melancholic and atypical symptoms. A KQIDS-SR score of 16 or more represents significant depression. The STAI (41) comprises two sets of 20 items each with a 4-point Likert scale to assess state anxiety (STAI-S) and trait anxiety (STAI-T). Only STAI-S was used in this study, and a STAI-S score of over 62 indicates significant anxiety. If participants were identified with depression and/or anxiety, they were assessed by trained psychiatrists or psychologists to screen the states of depression and/or anxiety through objective verification. In this study, the 17-item HRSD (42, 43) and the 14-item HAS (44, 45) were used. Total scores on the HRSD range from 0 to 54 points, which were classified into four categories: absence of depression (0 to 7), mild depression (8 to 16), moderate depression 17 to 23, and severe depression (above 24). The HAS scores range from 0 to 56, with 0 to 7 indicating absence of anxiety, 8 to 14 indicating mild anxiety, 15 to 23 indicating moderate anxiety, and 24 to 56 indicating severe anxiety.

### 2.3. Heart rate variability

Participants categorized into the four groups through clinical assessments underwent ECG recording while in a seated position in a quiet and comfortable room. The ECG signals were recorded at 500 Hz (SA-3000P, Medicore Co., Ltd., Seoul, Korea) for 5 min to reliably estimate HRV indices from the acquired signals (24, 46–49). The HRV indices were analyzed using MATLAB 2017b (Mathworks, Inc., MA, United States). At first, raw ECG signals were manually inspected to ensure that they do not contain any unnecessary components such as motion artifacts. After artifact correction, approximately 5–10% of R peaks were discarded from the signals. From artifact-free ECG signals, the electrocardiographic R-peaks used to derive RR intervals were clearly detected by applying peak detection algorithms. Finally, the defined RR intervals were used to calculate several HRV indices (21, 24) as follows: the time domain indices (50)—mean heart rate (Mean HR), mean of all RR intervals (Mean RR), SDRR, standard deviation of differences between adjacent RR intervals (SDSD), RMSSD, and percentage of differences between adjacent RR intervals >20 ms (pNN20); frequency domain indices (51)—normalized HF (nHF, 0.15 to 0.4 Hz), normalized LF (nLF, 0.04 to 0.15 Hz), and LF/HF. The time domain indices of HRV can estimate overall changes in HRV, whereas the frequency domain indices of HRV can estimate the modulation of sympathetic and parasympathetic nervous systems or their balance.

### 2.4. Statistical analysis

To compare the differences in demographics and clinical characteristics, independent t-tests (for continuous variables) and chi-square tests (for categorical variables) were performed between groups 1 and 2, and between groups 3 and 4. Additional independent t-tests and Mann–Whitney U tests were conducted between the groups to determine how HRV indices differed depending on the discrepancies between clinician-rated and self-rated scores of depression or anxiety. To investigate the group differences in depth, effect sizes (ES) were calculated based on the results of the t-test. A random effects model was used to calculate the standardized mean differences. Likewise, to assess the effectiveness of self-rated measures, the differences in HRV indices were investigated using independent t-tests and Mann–Whitney *U* tests between patients who were not depressed or anxious and those who were rated as depressed or anxious through self-rated assessments. Moreover, Pearson’s correlation analysis was conducted to explore the relationship between HRV indices and both clinician-rated and self-rated measures of depression or anxiety. Most of the statistical analyses were performed using SPSS version 25 (IBM Corp., Armonk, NY, United States). The calculation of ES was conducted using STATA version 16.0 (StataCorp LLC., College Station, Texas, United States). For all the analyses, statistical significance was defined as a two-tailed value of *p* of ≤0.05.

## 3. Results

### 3.1. Demographical and clinical characteristics of the participants

As mentioned in the Methods section, participants were divided into each group based on the results of self-rated and clinician-rated measures used to assess the states of depression and anxiety. The final groups were constituted as follows: group 1 (*n* = 28), clinician-rated and self-rated depression; group 2 (*n* = 68), only self-rated depression; group 3 (*n* = 44), clinician-rated and self-rated anxiety; group 4 (*n* = 20), only self-rated anxiety. Groups 1 and 2 comprised participants with depressive symptoms and groups 3 and 4 comprised participants with anxious symptoms. The differences between the groups were that the depressive or anxious states of the participants in groups 2 and 4 were identified using self-rated questionnaires, whereas those in groups 1 and 3 were diagnosed by both self-rated and clinician-rated assessments. Additionally, groups A (no depression group, *n* = 40) and B (no anxiety group, *n* = 55) were constituted for further analysis among the excluded participants based on the cut-off values for each self-rated questionnaire.

Demographic and clinical characteristics of participants are presented in Table 1. As expected, there were no variables showing significant differences between groups except for the clinician-rated outcomes. The HRSD score of group 2 was lower than that of group 1 (*p* < 0.001), indicating that the patients in group 2 were not diagnosed as having severe depression by clinicians. Similarly, the HAS score of group 4 was lower than that of group 3 (*p* < 0.001), representing that the patients in group 4 were not diagnosed as having severe anxiety by clinicians. Meanwhile, the main type in the DSM-5 diagnosis category assigned to participants was depressive disorders (35.71%, *n* = 35), followed by trauma-and stressor-related disorders (30.61%, *n* = 30) including adjustment disorders, and anxiety disorders (15.31%, *n* = 15). Other categories included somatic symptom disorders (7.14%, *n* = 7) and substance use and addictive disorders (4.08%, *n* = 4).

**Table 1:** Comparisons of demographic and clinical characteristics between groups 1 and 2, and between groups 3 and 4.

Characteristics	Group 1	Group 2	*p*-value	Group 3	Group 4	*p*-value
	*n* = 28	*n* = 68		*n* = 44	*n* = 20	
Female, No. (%)	15 (53.57)	41 (60.29)	0.544	23 (52.27)	13 (65.00)	0.341
Age, mean (SD), years	33.96 (8.70)	33.03 (7.98)	0.613	32.73 (8.17)	34.35 (8.46)	0.469
HRSD scores, mean (SD)	27.43 (3.29)	16.85 (4.58)	**< 0.001**	–	–	–
KQIDS-SR scores, mean (SD)	25.57 (5.61)	24.03 (5.11)	0.195	–	–	–
HAS scores, mean (SD)	–	–	–	31.34 (5.67)	15.15 (6.31)	**< 0.001**
STAI-S scores, mean (SD)	–	–	–	69.20 (4.38)	69.10 (4.81)	0.932

Independent *t*-tests were performed to compare continuous variables; Chi-square test was used for the cross-analysis of categorical variable. Bold type indicates statistically significant *p*-values.

Group 1: clinician-rated and self-rated depression; Group 2: only self-rated depression; Group 3: clinician-rated and self-rated anxiety; Group 4: only self-rated anxiety. SD, standard deviation; HRSD, Hamilton Rating Scale for Depression; KQIDS-SR, Korean Quick Inventory of Depressive Symptomatology Self-Report; HAS, Hamilton Anxiety Scale; STAI-S, State–Trait Anxiety Inventory-State.

### 3.2. Correlations of HRV indices with depression and anxiety scales

Pearson’s correlation analysis was used to determine the relationship between HRV indices and clinical assessments for depression or anxiety. As shown in Table 2, the self-rated and clinician-rated assessments for depression were positively correlated (*r* = 0.214, *p* = 0.036); however, those assessments for anxiety were not significantly correlated (*r* = 0.017, *p* = 0.894). Several HRV indices showed significant correlations with only the clinician-rated assessments—HRSD and HAS. The HRSD score had positive correlations with Mean HR, nLF, and LF/HF (*r* = 0.262, *r* = 0.309, *r* = 0.313; *p* = 0.010, *p* = 0.002, *p* = 0.002, respectively) but had negative correlations with Mean RR and nHF (*r* = −0.239, *r* = −0.309; *p* = 0.019, *p* = 0.002, respectively). For the HAS score, there were positive correlations between Mean HR and nLF (*r* = 0.251, *r* = 0.258; *p* = 0.045, *p* = 0.040) but a negative correlation between nHF (*r* = −0.258; *p* = 0.040). The self-rated questionnaires, KQIDS-SR and STAI-S, did not show any significant correlations with the HRV indices.

**Table 2:** Correlations matrix between HRV indices and clinical measures (clinician-rated and self-rated report).

Variables	Depression states (*n* = 96)		Anxiety states (*n* = 64)	
	HRSD	KQIDS-SR	HAS	STAI-S
HRSD	1	**0.214** ^*^	–	–
KQIDS-SR	**0.214** ^*^	1	–	–
HAS	–	–	1	0.017
STAI-S	–	–	0.017	1
Mean HR (ms)	**0.262** ^**^	0.046	**0.251** ^*^	−0.036
Mean RR (ms)	**−0.239** ^*^	−0.050	−0.200	0.044
SDRR (ms)	−0.109	−0.002	−0.133	−0.030
SDSD (ms)	−0.138	−0.043	−0.147	−0.027
RMSSD (ms)	−0.138	−0.043	−0.147	−0.027
pNN20 (%)	−0.198	−0.071	−0.215	−0.030
nHF (nu)	**−0.309** ^**^	−0.052	**−0.258** ^*^	−0.209
nLF (nu)	**0.309** ^**^	0.052	**0.258** ^*^	0.209
LF/HF	**0.313** ^**^	0.025	0.234	0.185

Pearson’s correlation analysis was performed to assess the association between HRV indices and each questionnaire. Bold type indicates statistically significant *p*-values.

^*^p < 0.05, ^**^p < 0.01.

HRV, heart rate variability; HRSD, Hamilton Rating Scale for Depression; KQIDS-SR, Korean Quick Inventory of Depressive Symptomatology Self-Report; HAS, Hamilton Anxiety Scale; STAI-S, State–Trait Anxiety Inventory-State; Mean HR, mean heart rate; Mean RR, mean of all RR intervals; nHF, normalized high-frequency power; nLF, normalized low-frequency power; LF/HF, low- to high-frequency ratio.

### 3.3. Understanding the meaning of HRV indices of depressive or anxious symptoms

**Figure 2:**
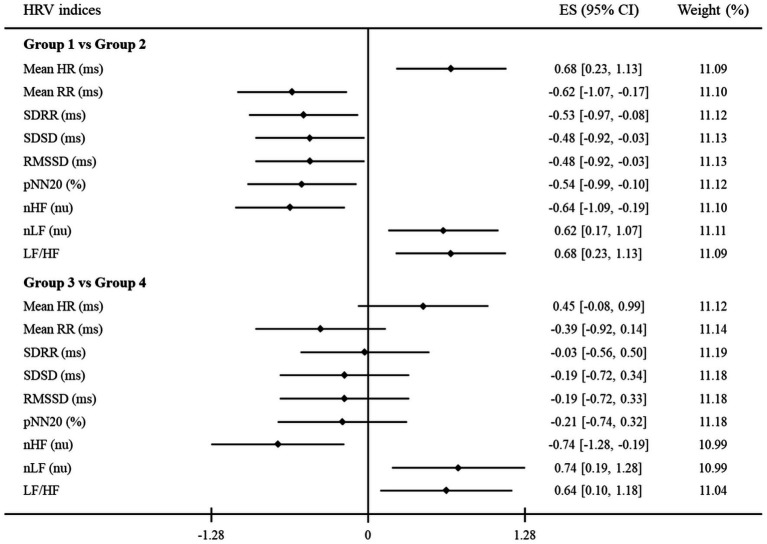
A forest plot showing the effect sizes (ES) and confidence intervals (CI) of HRV indices compared between groups. The vertical solid black line represents a mean difference of zero or no effect. Each black diamond shows the standardized mean difference. The upper and lower range of the line connected to the diamond shows the 95% CI for the ES. Group 1: clinician-rated and self-rated depression; Group 2: only self-rated depression; Group 3: clinician-rated and self-rated anxiety; Group 4: only self-rated anxiety. HRV, heart rate variability; Mean HR, mean heart rate; Mean RR, mean of all RR intervals; SDRR, standard deviation of all RR intervals; SDSD, standard deviation of differences between adjacent RR intervals; RMSSD, root mean square of successive RR interval differences; pNN20, percentage of differences between adjacent RR intervals >20 ms; nHF, normalized high-frequency power; nLF, normalized low-frequency power; LF/HF, low- to high-frequency ratio.

Table 3 represents the mean values of HRV indices for each group. The differences in HRV indices between groups 1 and 3 had a similar tendency to those between groups 2 and 4: increase in Mean HR, nLF, and LF/HF; decrease in Mean RR, SDRR, SDSD, RMSSD, pNN20, and nHF. Both the time and frequency domain HRV indices were significantly different between groups 1 and 2, whereas groups 3 and 4 only had significant differences in the frequency domain HRV indices. These results are depicted in Figure 2. The standardized mean difference (ES) and 95% confidence intervals (CI) between groups are represented in the forest plot. The vertical solid black line indicates a point where the ES is zero, which means that there are no significant differences between the groups. Between groups 1 and 2, Mean HR, nLF, and LF/HF indices had positive ES while all other indices had negative ES. The 95% CI of each HRV index between groups 1 and 2 did not include the point where ES is zero, but most of those between groups 3 and 4 did include that point. Similar to the results in Table 3, all HRV indices between groups 1 and 2 showed significant differences. In the same manner, only frequency domain HRV indices between groups 3 and 4 showed significant differences.

**Table 3:** Comparison of HRV indices between groups 1 and 2, and between groups 3 and 4.

HRV indices	Group 1	Group 2	*p*-value	Group 3	Group 4	*p*-value
	*n* = 28	*n* = 68		*n* = 44	*n* = 20	
Mean HR (ms)	88.08 ± 12.35	79.94 ± 11.69	**0.003**	85.87 ± 12.88	80.43 ± 9.39	0.105
Mean RR (ms)	695.50 ± 106.72	766.95 ± 116.39	**0.006**	714.69 ± 110.14	756.26 ± 93.38	0.148
SDRR (ms)	29.46 ± 10.75	38.38 ± 18.63	**0.045**	34.87 ± 17.05	35.32 ± 15.24	0.728
SDSD (ms)	18.17 ± 13.15	25.39 ± 15.68	**0.011**	20.62 ± 12.88	23.09 ± 11.71	0.385
RMSSD (ms)	18.15 ± 13.13	25.35 ± 15.65	**0.010**	20.59 ± 12.86	23.06 ± 11.70	0.377
pNN20 (%)	23.58 ± 24.43	36.82 ± 24.03	**0.011**	28.68 ± 23.89	33.64 ± 21.95	0.354
nHF (nu)	0.33 ± 0.20	0.45 ± 0.19	**0.006**	0.35 ± 0.19	0.49 ± 0.18	**0.006**
nLF (nu)	0.67 ± 0.20	0.55 ± 0.19	**0.006**	0.65 ± 0.19	0.51 ± 0.18	**0.006**
LF/HF	3.04 ± 2.12	1.81 ± 1.66	**0.006**	2.81 ± 2.08	1.53 ± 1.71	**0.006**

Values represent mean ± standard deviation. As all HRV parameters except Mean HR were not normally distributed, comparisons between groups were conducted using independent t-tests for a normally distributed Mean HR, and Mann–Whitney *U* tests for other variables. Bold type indicates statistically significant *p*-values.

Group 1: clinician-rated and self-rated depression; Group 2: only self-rated depression; Group 3: clinician-rated and self-rated anxiety; Group 4: only self-rated anxiety. HRV, heart rate variability; Mean HR, mean heart rate; Mean RR, mean of all RR intervals; SDRR, standard deviation of all RR intervals; SDSD, standard deviation of differences between adjacent RR intervals; RMSSD, root mean square of successive RR interval differences; pNN20, percentage of differences between adjacent RR intervals > 20 ms; nHF, normalized high-frequency power; nLF, normalized low-frequency power; LF/HF, low- to high-frequency ratio.

**Figure 3:**
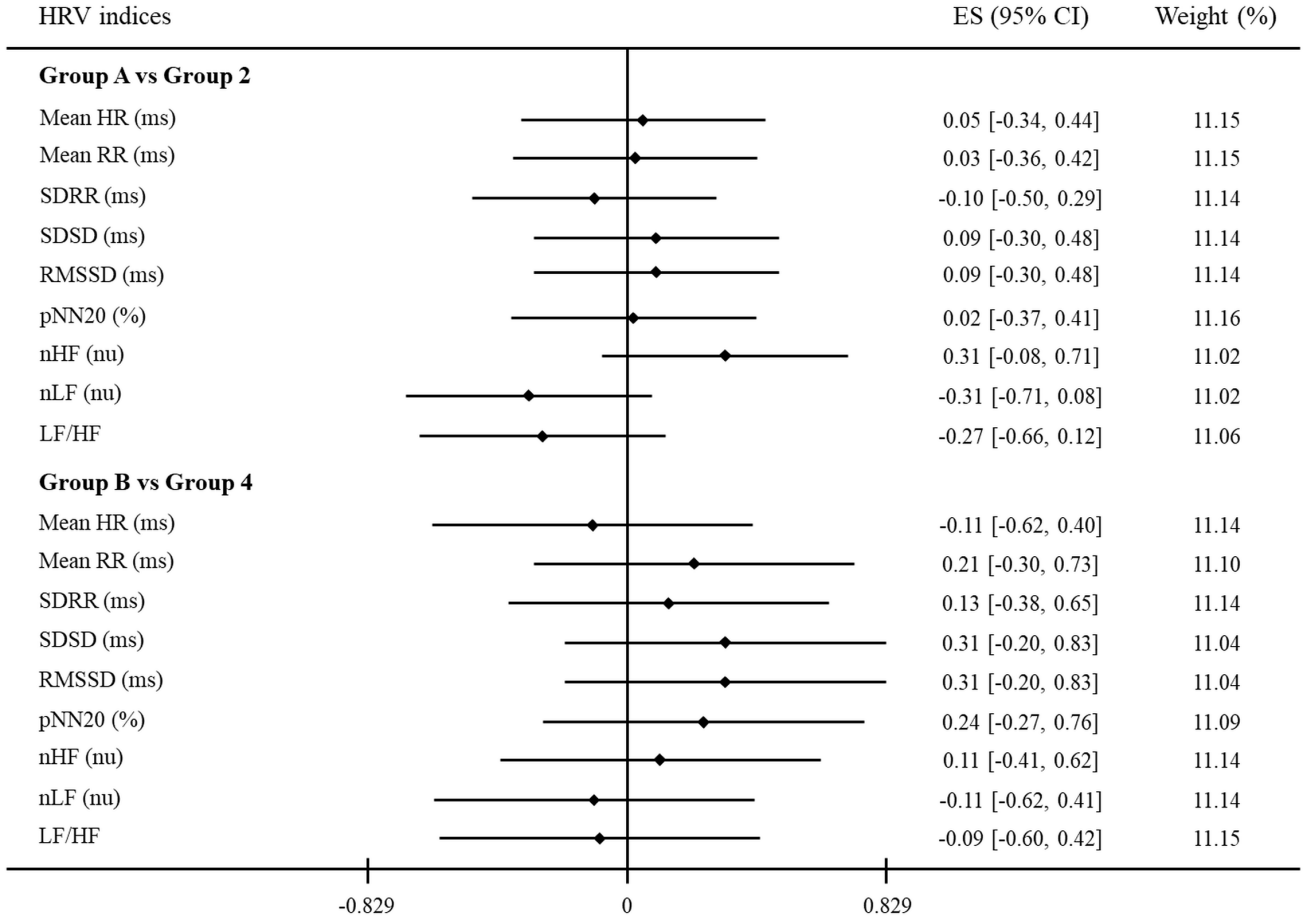
A forest plot showing the effect sizes (ES) and confidence intervals (CI) of HRV indices compared between groups. The vertical solid black line represents a mean difference of zero or no effect. Each black diamond shows the standardized mean difference. The upper and lower range of the line connected to the diamond shows the 95% CI for the ES. Group A: no depression group; Group 2: only self-rated depression; Group B: no anxiety group; Group 4: only self-rated anxiety. HRV, heart rate variability; Mean HR, mean heart rate; Mean RR, mean of all RR intervals; SDRR, standard deviation of all RR intervals; SDSD, standard deviation of differences between adjacent RR intervals; RMSSD, root mean square of successive RR interval differences; pNN20, percentage of differences between adjacent RR intervals >20 ms; nHF, normalized high-frequency power; nLF, normalized low-frequency power; LF/HF, low- to high-frequency ratio.

Furthermore, for the purpose of identifying another relationship between HRV indices and clinical assessments from a new perspective, Table 4 and Figure 3 are presented by applying the same method as above to other groups. Thus, new groups A and B of participants with no depression and anxiety were compared to groups 2 and 4, respectively, as mentioned earlier. As shown in Table 4, all HRV indices were not significantly different between groups A and 2 and groups B and 4. Similarly, in Figure 3, the 95% CI of all HRV indices contained the point where ES is zero. These results again revealed that there were no significant differences between groups A and 2 and groups B and 4.

**Table 4:** Comparison of HRV indices between groups A and 2, and between groups B and 4.

HRV indices	Group A	Group 2	*p*-value	Group B	Group 4	*p*-value
	*n* = 40	*n* = 68		*n* = 55	*n* = 20	
Mean HR (ms)	80.59 ± 14.74	79.94 ± 11.69	0.894	78.96 ± 14.34	80.43 ± 9.39	0.623
Mean RR (ms)	770.39 ± 148.49	766.95 ± 116.39	0.894	785.87 ± 149.25	756.26 ± 93.38	0.312
SDRR (ms)	36.60 ± 13.41	38.38 ± 18.63	0.844	37.48 ± 16.35	35.32 ± 15.24	0.765
SDSD (ms)	26.88 ± 16.27	25.39 ± 15.68	0.508	28.49 ± 18.52	23.09 ± 11.71	0.402
RMSSD (ms)	26.84 ± 16.24	25.35 ± 15.65	0.512	28.45 ± 18.48	23.06 ± 11.70	0.402
pNN20 (%)	37.32 ± 23.75	36.82 ± 24.03	0.934	39.64 ± 25.04	33.64 ± 21.95	0.415
nHF (nu)	0.51 ± 0.19	0.45 ± 0.19	0.132	0.51 ± 0.19	0.49 ± 0.18	0.710
nLF (nu)	0.49 ± 0.19	0.55 ± 0.19	0.132	0.49 ± 0.19	0.51 ± 0.18	0.710
LF/HF	1.39 ± 1.31	1.81 ± 1.66	0.142	1.40 ± 1.38	1.53 ± 1.71	0.710

Values represent mean ± standard deviation. As all HRV parameters except Mean HR, nHF, and nLF were not normally distributed, comparisons between groups were conducted using independent t-tests for a normally distributed Mean HR, nHF, and nLF, and Mann–Whitney *U* tests for other variables.

Group A: no depression group; Group 2: only self-rated depression; Group B: no anxiety group; Group 4: only self-rated anxiety. HRV, heart rate variability; Mean HR, mean heart rate; Mean RR, mean of all RR intervals; SDRR, standard deviation of all RR intervals; SDSD, standard deviation of differences between adjacent RR intervals; RMSSD, root mean square of successive RR interval differences; pNN20, percentage of differences between adjacent RR intervals > 20 ms; nHF, normalized high-frequency power; nLF, normalized low-frequency power; LF/HF, low- to high-frequency ratio.

## 4. Discussion

The present study evaluated the relationship between HRV and clinical evaluation results in subjects complaining of depressive and anxious symptoms. To increase the availability for clinical use of HRV indices, we sought to determine whether each HRV index is associated with objective or subjective implications of clinical measures. We extracted and utilized the indices of time and frequency domains of HRV reflecting ANS modulation. For clinical evaluations of depressive and anxious symptoms, both objective assessments by clinicians (HRSD and HAS) and subjective assessments by oneself (KQIDS-SR and STAI-S) were performed. As a result, it was confirmed that HRV indices changed significantly according to the severity of depressive and anxious symptoms in line with previous findings: increase in nLF and LF/HF; decrease in SDRR, RMSSD, pNN20, and nHF (27, 29). To our knowledge, few studies to date have simultaneously demonstrated the relationship between HRV, clinician-rated assessment, and self-rated assessment. In particular, various types of HRV indices that are difficult to substitute with each other were used in this study. Moreover, this is the first study to conduct the above analysis on a person who complains of depressive or anxious symptoms regardless of diagnosis at their outpatient clinic visit, rather than on those who have been diagnosed with a specific psychiatric disorder. Consequently, it is considered that HRV can enable estimating the depressive or anxious symptoms in an individual who reported those symptoms, regardless of a specific diagnosis. Additionally, HRV is simple and easy to perform, highly reproducible, and can be measured multiple times at a low cost compared to clinical measures (52, 53). Based on these advantages of HRV, it can be considered a valuable indicator that may have the potential for early detection of psychiatric symptoms and disorders.

The first major finding of this study is that HRV indices have objective implications. Each HRV index was found to be statistically significant when the depressive and anxious symptoms were assessed through both clinician-rated and self-rated assessments rather than self-rated assessment alone. Additionally, there were no significant differences in HRV indices when comparing those who were assessed as having no depressive or anxious symptoms through clinician-rated and self-rated measurements with those who were assessed as having these symptoms through only self-rated measurement. These results support the implication that depressive and anxious symptoms can be evaluated more objectively and accurately through clinician-rated measures and HRV than self-rated measures. In other words, it may be considered that HRV has diagnostic usefulness similar to clinician-rated measures, which show higher diagnostic performance than self-rated measures. Furthermore, statistically significant correlations were found only between HRV indices and clinician-rated assessments. The correlations between HRV indices and self-rated assessments also showed almost coherent tendencies, although they were not significant. These results further support our suggestion that HRV indices can be considered objective indicators similar to clinician-rated measures based on the objective view of experts, rather than self-rated measures based on subjective opinions. For this reason, to measure the symptoms of depression or anxiety more clearly and practically, it could be more effective to apply clinician-rated assessments and HRV indices, although many previous studies had proposed that self-rated measures are capable of evaluating those states (13–16, 35, 37).

The second major finding of this study is that HRV indices are likely to be better indicators of depressive symptoms than anxious symptoms. Through correlation analysis, significant correlations were confirmed between HRV indices and clinician-rated assessments for both depressive and anxious symptoms; however, a stronger relationship appeared in the assessments for depressive symptoms. In other words, observed depression showed high correlations with HRV indices and hence, it may be possible to objectively estimate the severity of depressive symptoms through the HRV indices. Moreover, a meta-analysis based on previous studies examining HRV indices related to depressive symptoms revealed that significant differences were observed in both time and frequency domain HRV indices (54). Our results were not only consistent with past findings but also revealed significant differences in both time and frequency HRV indices according to the observed depressive symptoms that were assessed objectively. Through these results, HRV is regarded as a potential and effective biomarker to objectively predict the severity of depressive symptoms. In contrast, the statistical power was relatively low or absent in our results for anxious symptoms. There are conflicting findings on whether the time or frequency domain HRV indices can reflect some significant changes according to the presence or severity of anxiety states (55). Several previous studies comparing the patients with anxious symptoms to the controls have suggested that the significant differences in HRV indices regarding the symptoms were presented only when a stressful situation such as specific stimuli (e.g., nervousness, tension, apprehension, and worry) was given, but not in resting state (56, 57). These results may indicate that the reactive response to anxiety is reflected in HRV, but significant changes in the resting state are hard to detect using HRV. Our findings also support that it is relatively difficult to estimate the severity of anxious symptoms through HRV in the resting state. Consequently, time domain HRV indices can be used to identify depressive symptoms more precisely. Similarly, although frequency domain HRV indices also appear to be more specific for identifying depressive symptoms, it is more reasonable to perceive that these indices can reflect a broad range of distress symptoms including anxiety.

The current findings should be interpreted with respect to some limitations. First, all groups were constituted using cut-off values that were already designated per each clinical measure. When forming the groups in this study, a high cut-off value was used as a reference because we tried to include only participants with clear clinical symptoms. For this reason, a few cases with relatively mild depressive or anxious symptoms may have been excluded. Second, only one type of clinical evaluation scale for depressive or anxious symptoms was used in this study. There are various types of clinical measures that reflect different aspects of verifying depressive or anxious symptoms (58, 59). As a result, the extent or severity of those symptoms may vary depending on the type of clinical measures. In this study, HAS and STAI were implemented, which are frequently used to evaluate anxious symptoms. The clinician-rated measure, HAS, mainly focuses on somatic symptoms, whereas the self-rated measure, STAI, is consisted of ambiguous items representing a generalized state of malaise (41, 44, 45, 60). The correlation between HAS and STAI may not be significant due to the discrepancies in composition. Conversely, it is suggested that the clinician-rated and self-rated assessments on depression, HRSD and KQIDS-SR, have high coexistence validity and similar sensitivity to symptom change (40). Taken together, it can be estimated that the results of this study could vary if other clinical measures are used to evaluate the symptoms of depression and anxiety. Therefore, exploring the relationship between different types of clinical measures and HRV indices in the same way is worth considering in the future. Third, we did not include the patients according to specific DSM diagnosis. It is noteworthy that we wanted to find the symptom-specific HRV features regardless of the diagnosis, with respect to the transdiagnostic dimensional approach, which is a developing concept accompanying the release of the DSM-5. Fourth, the impact of comorbidity was not considered when interpreting the differences in HRV according to depressive and anxious symptoms. It is generally known that anxious symptoms are known to be common comorbidities in people with depressive symptoms (61). For this reason, our findings should be interpreted carefully considering that many subjects with comorbidity with symptoms of depression and anxiety in this study. Further study is needed to identify the changes in HRV indices associated with pure depressive and anxious symptoms. Lastly, several statistical limitations may affect the generalizability of our findings. Although the correlation was significant, some correlation coefficients were not high. Moreover, we did not correct for multiple comparisons that may possibly result in Type-I errors. Hence, our findings should be interpreted with caution.

## 5. Conclusion

In summary, this study explored the relationship between HRV and clinical assessments of depressive and anxious symptoms. Particularly, a novelty of this study is that both clinician-rated and self-rated assessments were used to identify the relationship with HRV simultaneously. Moreover, another novelty is that the subjects were not limited to a specific group of mental disorders but included those who showed symptoms of depression or anxiety. Overall, our results suggest that HRV is an objective indicator to reflect the reduction in vagal tone associated with depressive or anxious symptoms, and is especially more effective in representing the state of depression and estimating the severity of depressive symptoms compared with anxious symptoms. This study’s findings contribute to the early detection of and interventions for depressive and anxious symptoms in the future.

## Data availability statement

The original contributions presented in the study are included in the article/Supplementary materials, further inquiries can be directed to the corresponding authors.

## Ethics statement

The studies involving human participants were conducted in accordance with the Declaration of Helsinki, and approved by the Institutional Review Board of Gangnam Severance Hospital, Yonsei University (protocol code 3-2022-0009 and 8th March 2022). Written informed consent for participation was not required due to the retrospective nature of the study.”

## Author contributions

JH, BL, and JO: conceptualization, validation, and writing—original draft preparation. JH and JO: methodology, writing—review and editing, and visualization. JH: software and formal analysis. JH, HEK, J-JK, J-HS, EK, JYP, and JO: investigation. J-JK, J-HS, EK, JYP, BL, and JO: resources. HEK, J-JK, J-HS, EK, JYP, and JO: data curation. BL and JO: supervision. J-JK, J-HS, EK, JYP, and JO: project administration. JO: funding acquisition. All authors contributed to the article and approved the submitted version.

## Funding

This work was supported by the Industrial Technology Innovation Program (no. 20012603, Development of Emotional Cognitive and Sympathetic AI Service Technology for Remote (Non-face-to-face) Learning and Industrial Sites) funded by the Ministry of Trade, Industry & Energy (MOTIE, Korea).

## Conflict of interest

The authors declare that the research was conducted in the absence of any commercial or financial relationships that could be construed as a potential conflict of interest.

## Publisher’s note

All claims expressed in this article are solely those of the authors and do not necessarily represent those of their affiliated organizations, or those of the publisher, the editors and the reviewers. Any product that may be evaluated in this article, or claim that may be made by its manufacturer, is not guaranteed or endorsed by the publisher.

## Supplementary material

The Supplementary material for this article can be found online at: https://www.frontiersin.org/articles/10.3389/fpsyt.2023.1124550/full#supplementary-material
