# Supplementary material for: Differential relationship of observer-rated and self-rated depression and anxiety scales with heart rate variability features
Source: Front Psychiatry. 2023 Apr 3;14:1124550. doi: 10.3389/fpsyt.2023.1124550 (PMC10109339; doi:10.3389/fpsyt.2023.1124550)
Supplement: Supplementary file 1 [file Table_1.DOCX]

Supplementary Material

**Supplementary Table 1.** Group division based on the clinical assessments for depressive symptoms.

| **Depressive symptoms**  **(n = 137)** | | **KQIDS-SR > 15** | |
| --- | --- | --- | --- |
|  |  | **No** | **Yes** |
| **HRSD > 23** | **No** | **40 (Group A)** | **68 (Group 2)** |
|  | **Yes** | 1 | **28 (Group 1)** |

To divide the patients into groups according to the degree of depressive symptoms, KQIDS-SR (self-rated questionnaire) and HRSD (clinician-rated assessment) were used. Cut-off values corresponding to overt depressive symptoms were applied respectively as follows: KQIDS-SR > 15, HRSD > 23. Groups 1, 2, and A were used to analyze the results of this study.

Group 1: clinician-rated and self-rated depression; Group 2: only self-rated depression; Group A: no depression group. KQIDS-SR, Korean Quick Inventory of Depressive Symptomatology Self-Report; HRSD, Hamilton Rating Scale for Depression.

**Supplementary Table 2.** Group division based on the clinical assessments for anxious symptoms.

| **Anxious symptoms**  **(n = 137)** | | **STAI-S > 61** | |
| --- | --- | --- | --- |
|  |  | **No** | **Yes** |
| **HAS > 23** | **No** | **55 (Group B)** | **20 (Group 4)** |
|  | **Yes** | 18 | **44 (Group 3)** |

To divide the patients into groups according to the degree of anxious symptoms, STAI-S (self-rated questionnaire) and HAS (clinician-rated assessment) were used. Cut-off values corresponding to overt anxious symptoms were applied respectively as follows: STAI-S > 61, HAS > 23. Groups 3, 4, and B were used to analyze the results of this study.

Group 3: clinician-rated and self-rated anxiety; Group 4: only self-rated anxiety; Group B: no anxiety group. STAI-S, State-Trait Anxiety Inventory-State; HAS, Hamilton Anxiety Scale.
